# Disease-specific characteristics of vascular cell adhesion molecule-1 levels in patients with peripheral artery disease

**DOI:** 10.1007/s00380-018-1315-1

**Published:** 2018-12-07

**Authors:** Christoph Edlinger, Michael Lichtenauer, Bernhard Wernly, Rudin Pistulli, Vera Paar, Christine Prodinger, Florian Krizanic, Marcus Thieme, Jürgen Kammler, Christian Jung, Uta C. Hoppe, P. Christian Schulze, Daniel Kretzschmar

**Affiliations:** 1 Division of Cardiology, Department of Internal Medicine II, Paracelsus Medical University of Salzburg, Salzburg, Austria; 2 Department of Internal Medicine I, Friedrich Schiller University Jena, Jena, Germany; 3 Department of Dermatology, Paracelsus Medical University of Salzburg, Salzburg, Austria; 4 Department of Cardiology, Caritas Clinic Pankow, Berlin, Germany; 5 1st Medical Department-Cardiology, General Hospital Linz, Johannes Kepler University School of Medicine, Linz, Austria; 6 Division of Cardiology, Pulmonology and Vascular Medicine, Medical Faculty, University Duesseldorf, Moorenstraße 5, 40225 Düsseldorf, Germany; 7 Department of Angiology/Cardiology/Diabetology, Medinos Kliniken Sonneberg, Sonneberg, Germany; 8 University Clinic of Internal Medicine I, Cardiology/Angiology, Friedrich Schiller University Jena, Am Klinikum 1, 07747 Jena, Germany

**Keywords:** Biomarker, Peripheral artery disease, ELISA, VCAM-1, Cardiovascular disease

## Abstract

Peripheral arterial disease (PAD) is one of the most common manifestations of systemic atherosclerosis. The prevalence of unrecognized PAD is high, leading to a lack of opportunity to detect subjects at a high risk for cardiovascular events. Inflammatory processes play an important role in the disease initiation as well as in the disease progression. Vascular cell adhesion molecule 1 (VCAM-1), a biomarker of endothelial dysfunction, appears to be an important mediator in inflammatory processes. Therefore, we hypothesized that in patients with PAD, circulating VCAM-1 might be elevated due to its function in mediating adhesion of immune cells to the vascular endothelium in the process of endothelial dysfunction and inflammation, and, therefore, applicable as a diagnostic biomarker. A total of 126 non-consecutive patients were enrolled in this study, of whom 51 patients had typical clinical manifestations of PAD and as controls 75 patients with no history of PAD or cardiovascular disease. All serum samples were obtained either during hospitalization or during out-patient visits and analyzed for VCAM-1 by the ELISA. Compared with controls, median levels of VCAM-1 were significantly elevated in patients suffering from PAD (953 vs. 1352 pg/ml; *p* < 0.001). Furthermore, VCAM-1 appeared to be highly discriminative for the detection of PAD (AUC = 0.76; CI 0.67–0.83). We could not observe dynamics related to increasing disease stages according to Rutherford classes in patients with apparent PAD. VCAM-1 was shown to be a potential discriminator and biomarker for the severity of systemic atherosclerosis. In a logistic regression analysis, VCAM-1 was robustly associated with the diagnosis of PAD, even after correction for clinically relevant cofounders (namely age, arterial hypertension, diabetes and LDL levels). Thusly, VCAM-1 might serve as a biomarker for PAD screening and detection.

## Introduction

One of the most common manifestations of atherosclerosis is peripheral artery disease (PAD), with a prevalence of around 10–25% in population aged over 55 years and an estimated total number of about 27 millions of affected patients within the industrialized world. PAD is considered to be an independent predictor of cardiac or cerebrovascular events and requires aggressive medical management [1–3]. A significant higher mortality of individuals suffering from PAD has been shown in the past [4, 5].

Since its first description by Brodie in 1846, a huge variety of pathophysiologic mechanisms on the development and progression of PAD have been described [6]. Over the last decades, many major clinical studies on pathogenesis of PAD focus on the role of inflammatory processes [7]. In patients with elevated inflammatory markers [e.g., CRP, lipoprotein (a), Interleukin-6 (IL-6) and soluble intercellular adhesion molecule-1 (SICAM-1)], an increased risk for the development of PAD could impressively be shown in the Edinburgh Artery Study [8]. Nevertheless, the role of inflammatory parameters is still not fully unraveled, especially when it comes to potential diagnostic use or therapeutic target for medical treatments.

Today, the paramount diagnostic tool for the assessment of PAD is the ankle–brachial index (ABI) as a non-invasive tool (ABI value ≤ 0.9 in PAD). It has been shown to be a strong indicator of generalized atherosclerosis and also as a risk factor for cardiovascular diseases in general; a value ≤ 0.90 has been shown to be associated with increased mortality [9]. The use of ABI has its limitations, including a lack of trained personnel and specialized equipment for performing proper ABI measurements in many primary practices (therefore not applied frequently in routine practice) and a high false-negative rate in patients with calcified vascular diseases, such as patients with diabetes and chronic kidney disease. This contributes to the fact that PAD still remains undiagnosed in many patients at risk, especially in earlier stages, which in turn leads to rates of “silent” PAD in up to 23% of patients [10, 11].

Since the diagnosis of PAD has therapeutic implications, targeting at reducing atherosclerotic vascular diseases and resulting in significant lowering of morbidity and mortality associated with PAD, early diagnosis and treatment of PAD are essential [12].

Therefore, the development of an additional/alternate diagnostic tool, particularly a biomarker-based screening test with a blood marker, is of great importance and seems appealing and feasible for the usage in PAD screening based at primary-care level. Additionally, biomarker levels may provide a more precise measurement of the extent of systemic atherosclerosis, an important determinant of the degree of functional impairment in PAD, in comparison to ABI. Further, biomarkers have already been shown to be of great help for screening, diagnostic assessment of disease severity and for follow-up and the assessment of prognosis in many other (cardiovascular) disease entities [13–19].

Over last 20 years, several inflammatory and humoral biomarkers have been identified and many studies tried to recognize a typical humoral profile or single marker as a predictor of PAD. However, many studies yielded conflicting results, and up to date, no single biomarker has been found to be a significant predictor of PAD and to be a sensitive marker for early diagnosis of the disease.

We hypothesized that vascular cell adhesion molecule 1 (VCAM-1, CD 106), a transmembrane molecule that was first described in 1989 independently by two different groups [20], and whose soluble form can be readily measured in blood, might be suitable as a candidate biomarker for PAD diagnosis.

In general, VCAM-1 is expressed within the luminal and lateral wall of endothelial cells during inflammatory processes. Within inflammation, it acts as a mediator of immune-cell adhesion to the vascular endothelium. The soluble form VCAM-1 has been shown to promote monocyte chemotaxis. Its association with numerous chronical diseases such as chronic heart failure and rheumatic could be shown in the past [21, 22].

Recently, an association of elevated soluble VCAM-1 levels with new onset of atrial fibrillation (AF) could be shown in a prospective, population-based cohort study with two decades of follow-up [23]. Griffin et al. could demonstrate an increasing risk for the development of AF in a population-based cohort study of older adults [24]. O’Neil et al. observed an increased risk for the development of atrial fibrillation and a consecutive increase risk for cardioembolic insults in patients suffering from PAD [25]. Together with alternate markers of inflammation, such as hsCRP and oxidative stress (e.g., MPO and NT-pro-BNP), VCAM-1 has been consistently associated with PAD severity.

Up to date, no convincing investigations to clarify the role of VCAM-1 as a single predictive and diagnostic biomarker for PAD and the severity of generalized atherosclerosis, to reduce the prevalence of unrecognized PAD, have been published in literature.

We, therefore, measured VCAM-1 levels in PAD patients and tried to foster our knowledge on the association between PAD and vascular/endothelial inflammation. We hypothesized that VCAM-1 might be a potential biomarker in the screening and detection process of PAD and might consecutively contribute to identify individuals at high risk of cardiovascular events or death.

## Materials and methods

### Patients and controls

In our present study, a total of 126 patients were enrolled; all were admitted to the Department of Internal Medicine I at the University hospital of Jena. 51 of these patients were diagnosed with PAD by clinical examination, apparative diagnostic (Doppler ultrasound) and functional tests (6-min walk test). 75 patients served as controls after exclusion of coronary artery disease and PAD. Patients suffering from any acute or chronic diseases, which we sought to might have a potential interaction with our hypothesis (recent history of decompensated heart failure or myocardial infarction, acute or chronic infections, malignant disease, autoimmune disease, hyperthyroidism) were excluded. Further criteria of exclusion were advanced kidney failure (glomerular filtration rate < 30 ml/min.) and intake of immunosuppressive drugs. All participants gave their informed written consent before being enrolled in this study. All participants in this retrospective single-center study were either recruited at the Department of Cardiology or at the department of dermatology. The study protocol underwent approvement of the Ethics Committee at the Friedrich Schiller University, Jena, Germany. The study was performed in accordance to the principles of the Declaration of Helsinki (2000) and Good Clinical Practice.

### Blood-samples/laboratory analysis

After a clean venous puncture in controlled haemostasis, blood samples were taken from all participants and analyzed for VCAM-1. All obtained serum samples immediately underwent storage at − 80°. Standard laboratory parameters (low density lipoprotein, LDL: mmol/l; triglycerides: mmol/l; high density lipoprotein, HDL: mmol/l; C-reactive protein, CRP: mg/l; creatinine: (µmol/l); creatinine kinase: (µmol/l); BUN: (mmol/l); and blood cell count) were kindly measured and provided by the Department of Clinical Chemistry at the University Hospital Jena. Serum levels were analyzed for VCAM-1 by a commercially available enzyme-linked immunosorbent assay (ELISA) kit. (Human VCAM-1 (CD106) ELISA Development Kit, PK-EL-60062D, PromoCell GmbH, 69126 Heidelberg, Germany). All measurements were performed according to the manufacturer`s instruction. In short, ninety-six-well microtiter plates were coated overnight with the capture antibody. After blocking of plates, serum samples and standard protein in different concentrations were added to the wells and incubated for 2 h. After a washing step, a biotin-labeled anti-body was added to each well and incubated once more. Then, plates were washed again and streptavidin–horseradish peroxidase (HRP) was added. A color reaction was achieved using tetramethylbenzidine (TMB; Sigma Aldrich, USA) and it was stopped by adding a sulphuric acid stop solution (Merck, Germany). Optical density values were measured at 450 nm on an ELISA plate reader (Bio-Rad Laboratories, Austria).

### Statistical analysis

GraphPad-Prism software (GraphPad-Software, La Jolla, CA, USA) and SPSS (22.0, SPSS Inc., USA) were used for all statistical analyses. Continuous data were expressed as mean ± standard error of the mean (SEM) and compared with student’s *T* test. VCAM-1 was expressed as medians (with interquartile ranges) and statistically analyzed using the Mann–Whitney *U* test. Differences between Rutherford stages were analyzed using the Kruskal–Wallis test with Dunn’s post hoc test. ROC analysis was performed and area under the curve (AUC) for the determination of the diagnostic accuracy of VCAM-1 for PAD was utilized. Univariable and multivariable logistic regression analysis was used to determine whether VCAM-1 was associated with the diagnosis of PAD. For the multivariable regression model, relevant confounders (age, arterial hypertension, type 2 diabetes and LDL-cholesterol levels) were included; then, a backward variable elimination was performed. Elimination criterion was a *p* value of more than 0.10. A *p* value of < 0.05 was considered as statistically significant. Furthermore, ROC analysis was performed and an optimal cut-off was calculated by the Youden Index (as described in Ref. [26]). The overall patient cohort was retrospectively divided into two groups: those above the optimal cut-off and those below this value.

## Results

Baseline characteristics are shown in Table 1. The Rutherford classification was used for disease staging. Patients within the control group were classified as Rutherford stage 0 (*n* = 55, 100%). Out of all patients, diagnosed with PAD, Rutherford stage 1 was present in 0%, Rutherford stage 2 in 25%, Rutherford stage 3 in 47%, Rutherford stage 4 in 16%, and Rutherford stage 5 in 12%. None of the patients had Rutherford stage 6. The majority of patients diagnosed with PAD were males (82% vs. 35%, *p* < 0.001). PAD patients were of significantly higher age than patients in the control group (62.92 ± 1.13 vs. 66.78 years ± 1.40; *p* = 0.04), their CRP levels were significantly elevated (2.28 ± 0.39 vs. 4.79 ± 1.23 mg/l, *p* = 0.03) at the time of hospital admission. Within the control group, median plasma levels of LDL (3.36 ± 0.12 vs. 2.58 ± 0.11 mmol/l; *p* < 0.001), HDL (1.45 ± 0.05 vs. 1.22 ± 0.04 mmol/l; *p* < 0.001) and triglycerides (1.51 ± 0.10 vs. 2.50 ± 0.36 mmol/l; *p* < 0.001) were significantly higher.

**Table 1:** Baseline characteristics of the study population (PAD, non-PAD patients and overall cohort)

	No PAD		PAD		*p* value
	Mean	SEM	Mean	SEM	
Age (years)	62.92	1.13	66.78	1.4	0.04
BMI	27.63	0.63	26.15	0.91	0.20
Ankle brachial index (ABI)	n/a		0.33	0.03	
CK (µmol/l)	2.08	0.16	1.97	0.2	0.67
Cholesterin (mmol/l)	5.55	0.14	4.8	0.14	0.00
LDL (mmol/L)	3.36	0.12	2.58	0.11	0.00
HDL (mmol/l)	1.45	0.05	1.22	0.04	0.00
LDL/HDL (ratio)	2.15	0.17	2.25	0.14	0.65
Triglycerides (mmol/l)	1.51	0.1	2.5	0.36	0.00
CRP (mg/l)	2.28	0.39	4.79	1.23	0.03
Thrombocytes (× 10^9^/l)	229.12	5.83	214.76	7.31	0.12
Leucocytes (× 10^9^/l)	7.08	0.17	7.42	0.2	0.20
Creatinine (µmol/l)	74.96	1.94	79.95	2	0.08
BUN (mmol/l)	5.7	0.22	5.46	0.3	0.52
Male	35%		82%		< 0.001
T2DM	18%		37%		0.01
Arterial hypertension	85%		90%		0.41
Fam. history for CVD	35%		18%		0.06
Smoking	30%		78%		< 0.001
Hyperlipidemia	50%		76%		0.01
Adipositas	45%		34%		0.023
Rutherford stage					
I	n/a		0%		
II	n/a		25%		
III	n/a		47%		
IV	n/a		16%		
V	n/a		12%		

Patients suffering from PAD were older, had lower LDL levels and had higher CRP levels

Major comorbidities in the PAD group were type 2 diabetes mellitus (18% in controls vs. 37% in PAD patients; *p* = 0.01), hyperlipidaemia (50% in controls vs. 76% in PAD patients; *p* < 0.01) and cigarette smoking (30% in controls vs. 78% in PAD patients, *p* < 0.001).

**Fig. 1:**
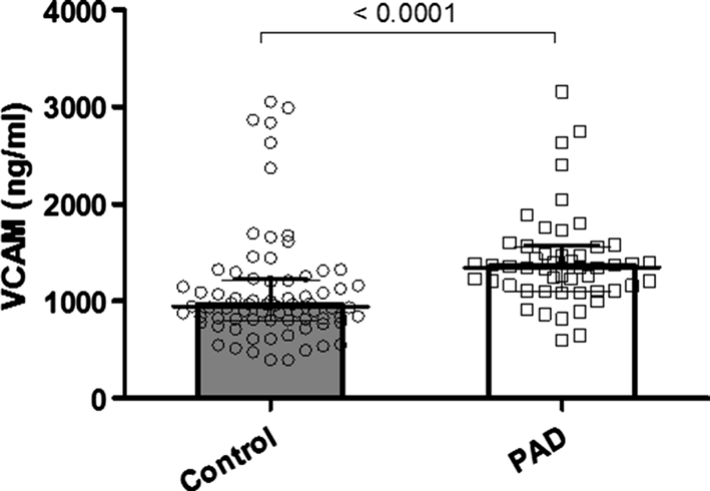
Comparison of VCAM-1 levels in PAD vs. non-PAD patients

**Fig. 2:**
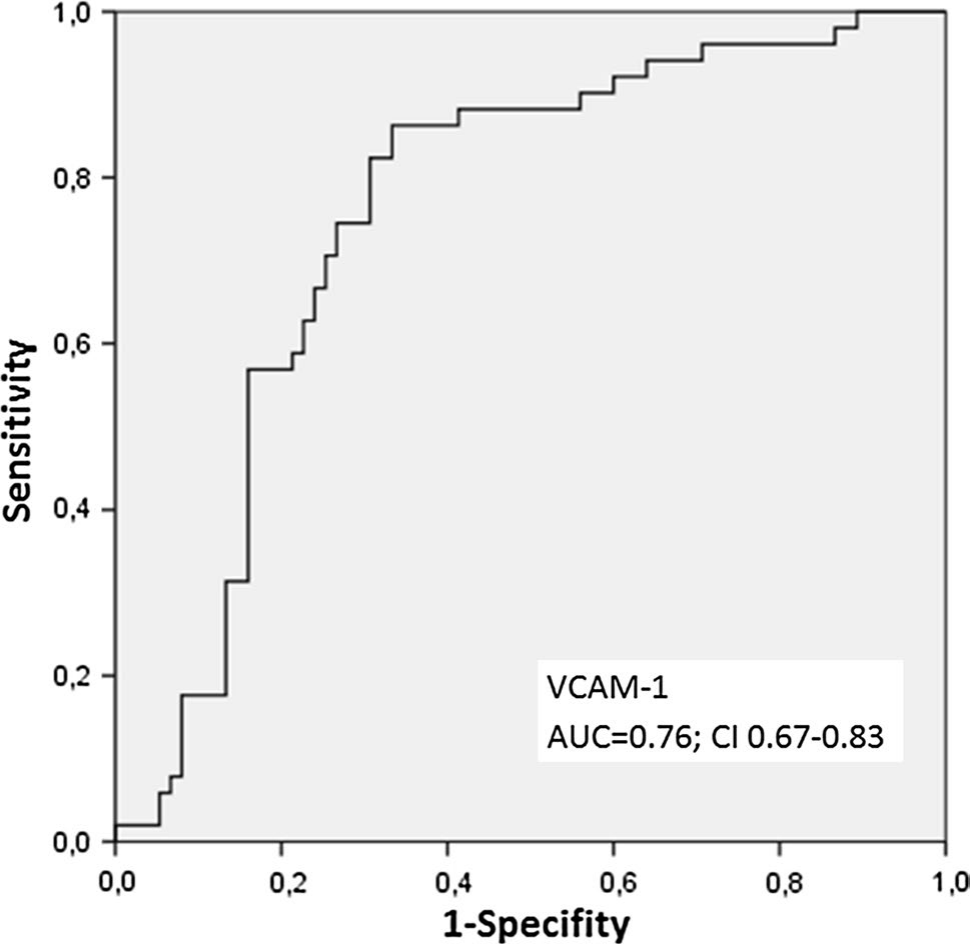
ROC curve for VCAM-1 for the diagnosis of PAD. Area under the curve (AUC) for VCAM-1 was 0.76; CI 0.67–0.83.44; *p* = 0.01

As shown in Fig. 1, VCAM-1 was significantly elevated in the PAD group (median 953 ng/ml in controls, IQR 812-1218 vs. 1352 ng/ml, IQR 1112-1569 in PAD patients, *p* < 0.0001). Within the PAD group, we could not observe a further rise in higher Rutherford stages. Median VCAM-1 levels according to Rutherford stage were: 1403 ng/ml in Rutherford II, 1342 ng/ml in Rutherford III, 1191 ng/ml in Rutherford IV and 1403 ng/ml in Rutherford Stage V (*p* = 0.74). VCAM-1 was highly discriminative for PAD in ROC analysis **(**AUC = 0.76; CI 0.67–0.83). By means of the Youden Index, we calculated an optimal cut-off for the detection of PAD with a VCAM-1 level of 1079 ng/ml (Fig. 2).

Patient characteristics for patients above and below the cutoff for VCAM-1 are shown in Table 2. Interestingly, patients with a VCAM-1 level below 1079 ng/ml had higher LDL levels (3.38 mmol/l vs. 2.73 mmol/l; *p* = 0.00). We could not observe relevant differences for HDL (1.41 mmol/l vs. 1.32 mmol/l; *p* = 0.20), LDL/HDL ratio (2.26 vs. 2.18 *p* = 0.60) and for triglycerides (1.69 mmol/l vs. 2.11 mmol/l; *p* = 0.21). Considering the fact that VCAM-1 is a well-known mediator of vascular inflammation, patients with VCAM-1 levels above our cutoff value of 1079 ng/ml had unsurprisingly higher CRP levels (3.34 mg/l vs. 1.91 mg/l; *p* = 0.02).

**Table 2:** Differences in patient characteristics between VCAM-1 levels below or above the calculated cut-off of 1079 ng/ml

	VCAM < 1079 (ng/ml)		VCAM > 1079 (ng/ml)		Overall cohort		*p* value
	Mean	SEM	Mean	SEM	Mean	SEM	
Patients (*n* =)	55		70				
Age (years)	62.69	10.40	65.98	8.51	64.39	9.57	0.07
BMI	27.03	4.60	27.46	6.07	27.23	5.31	0.69
Ankle brachial index (ABI)	0.32	0.24	0.33	0.18	0.33	0.19	0.91
Cholesterol (mmol/l)	5.55	1.17	4.97	1.06	5.23	1.15	0.00
LDL (mmol/l)	3.38	0.94	2.73	0.88	3.02	0.96	0.00
HDL (mmol/l)	1.41	0.37	1.32	0.39	1.36	0.38	0.20
LDL/HDL (ratio)	2.26	0.77	2.14	0.94	2.18	0.89	0.60
Triglycerides (mmol/l)	1.69	1.02	2.11	2.28	1.93	1.84	0.21
CRP (mg/l)	1.91	2.78	4.49	7.92	3.34	6.29	0.02
Thrombocytes (× 10^9^/l)	234	50	213	50	222	51	0.02
Leucocytes (× 10^9^/l)	7.39	1.41	7.05	1.49	7.20	1.46	0.21
Creatinine (µmol/l)	76.21	17.91	77.83	14.15	77.10	15.91	0.58
BUN (mmol/l)	5.65	1.66	5.55	2.09	5.59	1.89	0.78

Patients with VCAM-1 concentrations above our cut-off had higher CRP levels concentrations, but significantly lower total cholesterol and HDL levels

VCAM-1 was robustly associated with diagnosis of PAD in a logistic regression model (HR 1.001 95% CI 1.000–1.002; *p* = 0.01). Even after correction for clinically relevant cofounders (age, arterial hypertension, type 2 diabetes and LDL-cholesterol levels), VCAM-1 concentration [HF 1.001, 95% CI (1.0001–1.0018); *p* = 0.02] remained associated with the presence of PAD.

## Discussion

PAD is one of the most common manifestations of systemic arteriosclerosis, affecting about 10–25% of the general population. Due to the fact that PAD is very frequently asymptomatic, affected people often remain underdiagnosed and consecutively untreated [27]. Once PAD gets symptomatic, patients have a worse prognosis than individuals suffering from other forms of cardiovascular or cerebrovascular disease. Compared to coronary artery disease (CAD) and other cerebrovascular diseases, individuals suffering from PAD have the highest 1-year rate of atherothrombotic events [28]. The high prevalence of unrecognized PAD is largely attributable to diagnostic hurdles (e.g., infrequent use and/or false negative or increased ABI value in case of calcified vessels) that could be reduced with the introduction of specific biomarkers in screening algorithms.

The role of inflammatory processes in PAD development and PAD progression has impressively been shown in the past [7, 29]. Many current and ongoing clinical trials in PAD focus on endothelial inflammation. Vascular cell adhesion molecule 1 (VCAM-1), a transmembrane molecule acting as a mediator of immune cell adhesion to the vascular endothelium during inflammatory processes, has been shown to be associated in chronic heart failure and rheumatic disease [21, 22]. An association with smaller calf-muscle area (resulting in a poorer lower extremity performance) and lower calf-muscle strength in PAD patients [29] and with worse performance in 6-min walk test in PAD patients with higher soluble VCAM-1 concentrations has previously been shown [30].

As we expected and in consensus with similar studies [31, 32], VCAM-1 levels were significantly increased in individuals suffering from PAD compared to controls. Interestingly, we could not observe any further increase in VCAM-1 levels in higher Rutherford classes. Our potential explanation for this finding was that individuals in higher Rutherford stages are more likely to present with clinical symptoms. Once patients are diagnosed with symptomatic PAD, their individual risk factors are usually being treated, which might have an impact on inflammatory cytokines such as VCAM-1 [31]. Eventually even lifestyle modification such as weight loss or stop of cigarette smoking might be a potential explanation for the observed stagnation in VCAM-1 levels.

As the main finding of this current study, we could show for the first time in reliable statistics that VCAM-1 levels were highly discriminative for the detection of PAD. ROC analysis revealed that at a fold change cut-off of 1079 ng/ml, VCAM-1 was associated with a diagnostic accuracy of 76% in the prediction of PAD in our patients and significantly discriminate patients with PAD from those without PAD. Even after correction for clinically relevant cofounders (namely arterial hypertension, type 2 diabetes mellitus, age, LDL levels and cigarette smoking), VCAM-1 was still robustly associated with diagnosis of PAD in a logistic regression.

In the literature, the association of serum level of VCAM‐1 and diagnosis of PAD was not consistent; several studies were limited by a small number of participants (*n* < 50) [31, 33]. Strong evidence has been established for the association of higher levels of VCAM‐1 with a higher risk of PAD in patients with hemodialysis and those with diabetes [34]. A recent large study described, similar to our data, increased VCAM-1 levels in PAD patients alongside to several other inflammatory markers. However, no correlation with the clinical Rutherford stages and analysis of VCAM-1 regarding its value as a prospective biomarker has been performed [32]. We, therefore, show for the first time that VCAM-1 has potential to be used as a biomarker in the diagnosis of PAD, acting as an additive diagnostic tool to identify patients with PAD at an earlier stage of the disease thereby reducing the number of unrecognized PAD.

In the context of medical treatment, we could additionally observe an interesting finding concerning LDL-cholesterol levels. In our cohort, the optimal cut-off for the detection of PAD was 1079 ng/ml. Interestingly, individuals with a VCAM-1 level below 1079 ng/ml showed significantly higher LDL-cholesterol levels. In our opinion, this finding is primarily caused by the intake of statins, which are usually prescripted, once PAD is diagnosed. This effect has also been described in an experimental analysis by Xu et al. They conclusively showed that pitavastatin reduced VCAM-1 mRNA and protein expression in TNF-alpha stimulated cultured human umbilical vein endothelial cells (HUVECs) [33]. A plethora of previous studies provided evidence for further pleiotropic effects of statins beyond their cholesterol-lowering effects [35]. However, anti-inflammatory mechanism and effects on endothelial homeostasis of statins have not been fully elucidated yet [36]. Moreover, also the intake of antiplatelet drugs might have influenced VCAM-1 levels. Antiplatelet therapy is recommended in all patients with symptomatic PAD [9, 37, 38]; however, in subclinical PAD, the usefulness and clinical benefit for patients remain elusive.

Based on these findings, we postulate by reinforcing previous (smaller) studies that VCAM-1 might be a promising biomarker for the detection of PAD and might contribute to a new diagnostic approach in systemic atherosclerosis, having an impact on cardiovascular mortality. However, further prospective studies are required to evaluate the clinical practicability and define the true clinical applicability of inflammatory markers in PAD detection.

### Limitations

The largest limitation of this study is its single-center, sample size and retrospective layout. Due to its retrospective experimental design, we cannot provide prospective data on mortality. Further prospective studies are warranted to determine the prognostic potential of VCAM-1 as a biomarker for the diagnosis of PAD in clinical practice. Additionally, no follow-ups were performed, and therefore, the dynamic regulation of VCAM-1 in the progression of PAD could not be further assessed in this current analysis. Moreover, a possible influence of confounders such as medication on VCAM-1 levels would be of great interest; nevertheless, as no information on medication, e.g., statin intake was available, this influence cannot be ruled out. Despite these limiting factors, we believe that the current study shows the potential VCAM-1 for the diagnosis of PAD.
